# Development of an Ultrahigh-Performance Liquid Chromatography–Triple Quadrupole Mass Spectrometry Method for Multiclass Phytohormone Quantification Via Multifunctional Chemical Derivatization

**DOI:** 10.1021/acs.jafc.6c01953

**Published:** 2026-05-18

**Authors:** Michael Gigl, Michael Dankesreiter, Larissa Barl, Carlos Agius, Christian Schmid, Magdalena Holzer, Chris-Carolin Schön, Claus Schwechheimer, Corinna Dawid

**Affiliations:** † Junior Research Group Food Processing and Health, ZIEL Institute for Food and Health, 9184 Technical University of Munich, Lise-Meitner-Str. 34, 85354 Freising, Germany; ‡ Professorship for Chemosensory Food Systems, TUM School of Life Sciences, Technical University of Munich, Lise-Meitner-Str. 34, 85354 Freising, Germany; § Chair of Plant Breeding, TUM School of Life Sciences, Technical University of Munich, Liesel-Beckmann-Str. 2, 85354 Freising, Germany; ∥ Chair of Plant Systems Biology, TUM School of Life Sciences, Technical University of Munich, Emil-Ramann-Strasse 8, 85354 Freising, Germany; ⊥ Chair of Food Chemistry and Molecular Sensory Science, TUM School of Life Sciences, Technical University of Munich, Lise-Meitner-Str. 34, 85354 Freising, Germany; # Leibniz Institute for Food Systems Biology at the Technical University of Munich, Lise-Meitner-Str. 34, 85354 Freising, Germany

**Keywords:** phytohormones, LC-MS/MS, plant stress, multifunctional chemical derivatization

## Abstract

Plant growth regulation and responses to biotic and abiotic stress factors are mediated by phytohormones. Understanding the effects of the phytometabolome is essential for addressing future global agricultural and food sector challenges. Therefore, a liquid chromatography–mass spectrometry method was developed to improve the ability to determine the concentrations of various phytohormones. Using only 20 mg of plant material, a total of 27 hormones from different classes, including 18 gibberellins, abscisic acid, salicylic acid, auxin, and jasmonates, can be analyzed in 10 min via the stable isotope dilution assay approach. Using a carbodiimide-hydrazine one-pot multifunctional chemical derivatization approach, the analytes can be detected with quantitation limits ranging from 0.04 to 29.9 nM. Application of the method to tomato, maize, and cress verified its applicability, thus enabling quantitative multiclass phytohormone profiling.

## Graphical abstract

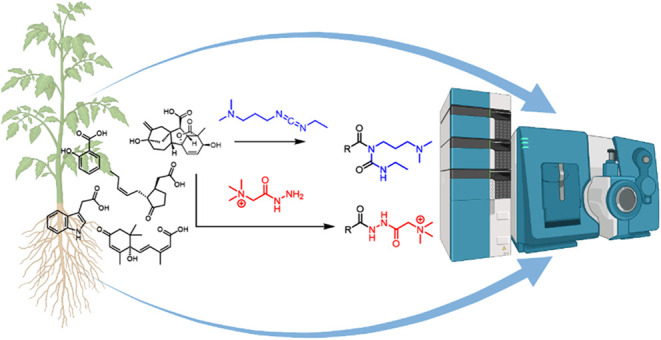

## Introduction

Plants must respond to various external stimuli, adapting their growth and development to changing environmental conditions such as temperature fluctuations, water and nutrient availability, and pathogen or herbivore activity.^1–3^ Adaptations to stress challenges and growth regulation in plants are controlled by phytohormones, which play a crucial role in many aspects of plant development.^4–6^ Phytohormones encompass a wide range of heterogeneous compound classes, including gibberellins (GAs),^7–9^ abscisic acid (ABA),^10,11^ jasmonates,^12–14^ salicylates,^15,16^ and auxins.^17–19^ Within these categories, various precursors, catabolites, and glycosylated storage forms further contribute to the chemical diversity of plant hormones.^20–23^ For example, GAs are a class of 136 known compounds found in plants and fungi, derived from the diterpene geranylgeranyl diphosphate.^7,24^ GAs promote growth in stems and leaves, influence root development and seed germination, and play a role in the flowering process of some plant species.^7,25^ Other major phytohormones include ABA, a sesquiterpenoid important for regulating seed germination and plant growth in response to external stress, such as high salt concentrations and drought.^10,26,27^ ABA can be temporarily inactivated by forming a glycosyl ester (ABA-Gluc), enabling its transport and storage. ABA is further metabolized through oxidation to phaseic acid and subsequent reduction to dihydrophaseic acid.^27^ In addition to their inherent bioactivity, phytohormones have important synergistic and antagonistic effects. Depending on their concentration, they can influence the biosynthesis of other hormones or modulate their effects. Salicylic acid (SA), for example, plays a crucial role in stress responses during water shortage, salt stress, and extreme temperatures and is involved in the action and regulation of other phytohormones.^16,28^ In wheat seedlings subjected to salt stress, the exogenous application of SA results in the accumulation of ABA and 3-indoleacetic acid (IAA).^16^ SA also has a regulatory effect on the hormones jasmonic acid (JA) and ethylene, where the accumulation of SA suppresses the biosynthesis of JA, and this antagonistic effect is further modulated by ethylene in *Arabidopsis thaliana*.^29^

Because the physiological effects of phytohormones are influenced by their endogenous concentrations, research on their functions and regulatory roles relies heavily on their accurate quantification in different plant tissues. However, phytohormones are produced in very low concentrations by plants, necessitating sensitive analytical techniques. Previously, the analysis of phytohormones often required large sample amounts and elaborate prefractionation methods or was focused on a very limited number of highly abundant analytes. Given the numerous representatives in each phytohormone class and their significant interactions, high-throughput methods are essential.^30^ Therefore, ultrahigh-performance liquid chromatography-tandem mass spectrometry (UHPLC-MS/MS) is the state-of-the-art platform used for the efficient separation and detection of phytohormones. Using UHPLC, even structurally similar analytes can be separated and detected with the high selectivity and sensitivity of the MS/MS systems. In recent years, significant advancements have been made in phytohormone profiling. Cai et al. developed a technique to analyze 54 phytohormones and successfully detected 36 of them in a 50 mg rice sample.^31^ Similarly, Luo et al. quantified 16 phytohormones from 100 mg *Brassica napus* *L*. flowers, whereas studies by Šimura et al. quantified 43 phytohormone-related analytes in *Arabidopsis* seedlings.^32,33^

However, detecting and quantifying phytohormones such as GAs in relevant concentrations remains challenging owing to their poor ionization efficiency in mass spectrometry (MS), which severely limits comprehensive phytohormone profiling. Therefore, chemical derivatization techniques were implemented to enhance the sensitivity of poorly ionizable compounds.^34^ For example, Li et al. achieved near-attomolar levels of GA following derivatization.^35,36^ A combination of different derivatization reagents has already shown promising results for the simultaneous determination of carboxylated hormones and brassinosteroids.^37^

Therefore, the present study utilizes a combination of multifunctional chemical derivatization with UHPLC-MS/MS and SIDA to enable multiclass hormone quantification, addressing the prevalent issue of poor ionization efficiency of certain phytohormones, particularly gibberellins (GAs), in MS detection. By derivatizing carbonyl groups GA and non-GA hormones simultaneously using a carbodiimide coupling reagent and a hydrazine-based quaternary amine reagent, the MS response for poorly ionizable hormones is enhanced. Thereby, hormone analysis was improved to a 10 min run time and expanded the range of analytes that can be quantified in small sample amounts, important for plant stress studies and to complement breeding studies on a molecular level.

## Materials and Methods

### Chemicals

The following phytohormone reference compounds and isotope-labeled standards were obtained commercially: GA1, GA3, GA4, GA5, GA6, GA7, GA8, GA9, GA13, GA14, GA15, GA19, GA20, GA34, GA44, GA51, GA53, GA95; 2-cis, 4-trans-abscisic acid, (±)-cis,trans-abscisic acid glucosyl ester; SA glucoside; GA1-d_2_, GA4-d_2_, GA5-d_2_, GA6-d_2_, GA7-d_2_, GA8-d_2_, GA9-d_2_, GA19-d_2_, GA20-d_2_, GA34-d_2_, GA44-d_2_, GA51-d_2_, GA53-d_2_; 2-cis-4-trans-abscisic acid-*d*_6_; SA-*d*_4_ were obtained from OlChemIm, (Olomouc, Czech Republic). IAA, salicylic acid, and IAA-*d*_2_ were purchased from Sigma-Aldrich (Schnelldorf, Germany), (±)-JA from Santa Cruz Biotechnology (Dallas, TX), (±)-JA-*d*_5_ from Cayman Chemicals (Ann Arbor, MI), phaseic acid, and dihydrophaseic acid from MedChemExpress (Sollentuna, Sweden).

Solvents used for the UHPLC-MS/MS analysis were of LC-MS grade (Honeywell, Seelze, Germany). Formic acid, pyridine, *tert*-butyl methyl ether (TBME), *N*-(3-(dimethylamino)­propyl)-*N′*-ethylcarbodiimide hydrochloride (EDC), diisopropylcarbodiimide (DIPC), diphenylurea, 1-phenyl-3-(3-(dimethylamino)­propyl)­urea, triethylamine (NEt_3_), triphenylphosphine, iodine, pyridinium chloride, *p*-tosyl chloride (*p*-TsCl), dichloromethane (DCM), and deuterated solvents (acetonitrile-d_3_, methanol-d_4_, and D_2_O) used for quantitative ^1^H nuclear magnetic resonance (qHNMR) spectroscopy were purchased from Sigma-Aldrich (Steinheim, Germany). 1,3-Bis­(3-(dimethylamino)­propyl)­urea was obtained from Synthonix, Inc. (Wake Forest, NC), and di*tert*-butylcarbodiimide (DTBC) was purchased from TCI Deutschland GmbH (Eschborn, Germany). Deionized water used for all experiments was prepared using a Milli-Q Advantage A10 water purification system (Millipore, Schwalbach, Germany).

### Plant Material

#### Tomato Samples

Tomato seeds (*Solanum lycopersicum* cultivar M82) were germinated on wetted filter paper at 23 °C in long day conditions (16:8 h light/night) after vernalization for 2 days at 4 °C in the dark. After cotyledon opening, the germinated M82 seeds were transferred to 3.6 × 3.6 cm rockwool cubes (Grodan, Industrieweg, The Netherlands). Two weeks after germination, the seedlings were supplemented with a nutrient solution containing 14.0 mM NO_3_^–^, 1.0 mM NH_4_^+^, 1.3 mM PO_4_^3–^, 7.8 mM K^+^, 5.0 mM Ca^2+^, 1.0 mM Mg^2+^, 2.74 mM SO_4_^2–^, 30.08 μM H_3_BO_3_, 1.0 μM MoO_4_^2–^, and EDTA-complexes of 35.0 μM Fe^3+^, 10.06 μM Mn^2+^, 5.01 μM Zn^2+^, and 1.0 μM Cu^2+^ (Merk Chemicals GmbH, Darmstadt, Germany). The tomato seedlings were further grown and maintained at 23 °C in long day conditions (16:8 h light/night) under 400 μmol m^–2^ s^–1^. Twenty-one days after germination true leaves of tomato seedlings were immediately snap-frozen in liquid nitrogen and stored at −80 °C until sample preparation in triplicate. Tomato seeds were harvested from tomato plants (*Solanum lycopersicum* cultivar M82) which were grown in a climate-controlled greenhouse located in Plant Technology Center in Dürnast, Freising, Germany (48°24′21.5″ N 11°41′28.5″ E). The tomato seedlings were germinated and grown in 3.6 × 3.6 cm rockwool cubes as specified before and then transferred to 10 × 10 × 6.5 cm rockwool blocks (Grodan, Industrieweg, The Netherlands). The seedlings were then transferred to the greenhouse into 15 L pots filled with 2–6 mm perlite (PERLIGRAN Extra, Knauf Aquapanel GmbH, Dortmund, Germany). Tomato seeds were then harvested from red ripe tomato fruits by cutting the tomato fruits in half, extracting the pulp and seeds into a cylinder, and mixing the mixture with 3 N HCl in a 1:1 ratio. After 30 min, seeds were collected by passing the solution through a sieve. The seeds were then further incubated in 10% (m/v) Na_3_PO_4_ for 30 min, washed with water through a sieve, and left to dry overnight on paper towels. The dried tomato seeds were then stored in paper bags at room temperature for 2 to 6 weeks. After storage, seeds were directly used for sample preparation in triplicate.

#### Maize Samples

Maize plants were cultivated in a growth chamber at 25 °C/20 °C day/night, 65% relative humidity, 16:8 h light/night, and 800 μmol m^–2^ s^–1^ photosynthetically active radiation until they reached the developmental stage V3.^38^ The developmental stages of maize were determined according to https://www.pioneer.com/us/agronomy/staging_corn_growth.html. For sampling, 1 cm segments of the fifth leaf were collected from two plants and pooled to generate each sample. Samples were immediately snap-frozen, homogenized in liquid nitrogen, and stored at −80 °C until sample preparation in triplicate.

#### Cress Samples

Cress seeds and plants (*Lepidium sativum*) were purchased from Frankonia Handels GmbH & Co. KG (Rottendorf, Germany) and Elmar Gimperlein (Albertshofen, Germany). Cress plants were dissected into leaves, stems, and roots and pooled to reach 20 mg (3–5 plants) for the workup procedure. Seeds were directly used for sample preparation in triplicate.

### Synthesis of Carbodiimide Derivatization Reagents

#### Synthesis of 1,3-diphenylcarbodiimide (DPC)

The procedure was performed according to Duangkamol et al. with minor modifications. In a flask, triphenylphosphine and I_2_ (1 mmol each) were combined with 10 mL of DCM and sonicated for 2 min using an ultrasonic bath. Then, NEt_3_ (2 mmol) and a suspension of 1,3-diphenylurea (1 mmol) in DCM (10 mL) were added to the resulting solution. The reaction was conducted under an argon atmosphere with sonication at room temperature and was completed in 10 min, indicated by yellow coloration.^39^ The product was dried by removing the solvent under a nitrogen stream, and the residue was extracted three times with *n*-hexane. The combined extracts were concentrated under nitrogen and purified via solid-phase extraction silica cartridges (5 g, 20 mL, Strata SiOH, Phenomenex). The structure of DPC was characterized using NMR spectroscopy: ^1^H NMR (400 MHz, CDCl_3_): δ [ppm] 7.22–7.17 (m, 4H, H-2–6, H-2′-6′), 7.38–7.32 (m, 6H, H-3–5, H-3′-5′).

#### Synthesis of 1,3-bis­(3-(dimethylamino)­propyl)­carbodiimide (DAPC)

The compound was synthesized according to Appel et al. with modifications. A mixture of the bis-1,3-(3-(dimethylamino)­propyl)­urea (5.2 mmol) and NEt3 (26.6 mmol) in DCM (10 mL), which was prepared and cooled in an ice bath. Subsequently, 20 mL of *p-*TsCl solution (11.4 mmol, in DCM) was slowly added with stirring.^40,41^ The resulting mixture was allowed to reach room temperature and then heated for 3 h under reflux. Subsequently, the reaction mixture was cooled to room temperature and extracted three times with potassium carbonate solution (40% w/v, 10 mL), and the resulting precipitate was filtered and washed with DCM. The organic phase was freed of solvent under reduced pressure using rotary evaporation. The crude product was then extracted five times with 10 mL TBME. The combined ether solutions were freed of solvent on the rotary evaporator, and a clear, brown, oily liquid was obtained. To achieve further purification, 500 mg of the product was combined with pyridinium chloride (2.0 mmol) in DCM (4 mL) and stirred for 5 min at RT. By adding MTBE (40 mL), the product was precipitated as the respective hydrochloride. The solvent mixture was removed and subsequently recrystallized from DCM and TBME. DAPC was structurally characterized by NMR spectroscopy. ^1^H NMR (500 MHz, CDCl_3_): δ [ppm] 1.75–1.64 (m, 4H, H-2, H-2′), 2.20 (s, 12H, H-4–5, H-4′-5′), 2.31 (t, *J* = 7.0 Hz, 4H, H-3, H-3′), 3.25 (t, *J* = 6.9 Hz, 4H, H-1, H-1′).

#### Synthesis of 1-phenyl-3-(3-(dimethylamino)­propyl)­carbodiimide (PDC)

The synthesis and workup were performed as described for DAPC, starting with a mixture of 1-phenyl-3-(dimethylamino)­propylurea (3.30 mmol) and an excess of NEt_3_ (16.0 mmol) and *p*-TsCl (3.84 mmol).^41^ PDC was structurally characterized by NMR spectroscopy: ^1^H NMR (500 MHz, MeOD): δ [ppm] 2.19–2.22 (m, 2H, H-2), 2.27 (s, 6H, H-4–5), 2.40 (t, 2H, H-3), 3.23 (t, *J* = 6.8 Hz, 2H, H-1), 7.22–7.34 (m, 5H, H-2′- 6′).

### Preparation of Stock Solutions

Reference compounds of all analytes and deuterated internal standards were accurately weighed and dissolved in MeOD-*d*_3_ (1 mL). Aliquots (600 μL) of all compounds were placed in NMR tubes, and the exact concentration of the individual stock solutions was determined by qHNMR, as reported in the literature.^42^ From the individual stock solutions, a reference mixture containing all unlabeled GAs (10 μM each), another reference mixture containing all other unlabeled phytohormones (50 μM each), and an internal standard mixture containing all stable isotope-labeled phytohormones (1 μM each) were prepared in acetonitrile/water (1:1 v/v).

### Optimization of Chemical Derivatization

#### Selection of Derivatization Reagents

The following derivatization reagents were tested for applicability in multifunctional chemical derivatization: Girard’s reagent T (GT) for non-GA hormones as well as the carbodiimides EDC, DIPC, DTBC, DPC, DAPC, and PDC for GAs.

#### Selection of Gibberellin Reagent

To optimize the derivatization procedure, different carbodiimides (EDC, DIPC, DTBC, DPC, DAPC, DAPC-HCl, and PDC) were used to prepare 120 mM solutions in EtOH, and a GA model mixture containing GA3, GA13, and GA19 (5 μM each) was prepared. Then, the GA model mixture (20 μL) was transferred into microcentrifuge tubes and evaporated using a stream of nitrogen. Then, a carbodiimide solution (20 μL, 120 mM) was added. Samples were placed in a thermoshaker at 40 °C and 600 rpm for 80 min. The resulting solutions were freed of solvent, and the residues were resuspended in ACN/H_2_O (1:1 v/v, 50 μL). Aliquots (5 μL) were injected into the UHPLC-MS/MS system, and peak areas and intensities of derivatized GAs were compared to determine the optimal derivatization reagent.

#### Optimization of Derivatization Temperature

The GA mixture was derivatized in triplicate with an ethanolic EDC-HCl solution containing 6% pyridine (120 mM, 20 μL) and placed on a thermoshaker for 80 min at temperatures of 10 °C, 25 °C, 40 °C, and 60 °C. The resulting solutions were freed of solvent, and the residue was resuspended in ACN/H_2_O (1:1 v/v, 50 μL) and injected into the UHPLC-MS/MS system. The respective peak areas and intensities were evaluated to determine the optimal derivatization temperature.

#### Optimization of Derivatization Time

Samples of the GA mixture were analyzed in triplicate and, after adding the EDC derivatization reagent (20 μL, 120 mM), incubated for 40, 80, 120, 180, 240, 300, and 420 min at 25 °C on a thermoshaker. After removing the solvent, ACN/H2O (1:1 v/v, 50 μL) was added, and the samples were analyzed by UHPLC-MS/MS. The resulting peak areas and intensities were determined, and the optimal derivatization time was identified.

#### Multifunctional Chemical Derivatization

To achieve a one-pot derivatization approach, the combination of a carbodiimide (EDC) and a hydrazine (GT) was chosen, as this combination is readily compatible under the same reaction conditions.^43,44^ The parameters were optimized for GA detection since these analytes were more challenging to accurately quantify at physiologically relevant concentrations compared to the higher abundant non-GA phytohormones. To verify this, derivatization under optimized conditions was performed with and without GT, and the UHPLC-MS/MS analysis showed that the resulting peak areas of GAs were not affected by the addition of GT, whereas the non-GA hormones were detected as GT derivatives (Table S1). Concentrations of EDC and GT were adapted from previous reports.^43^

### Sample Preparation

Plant material was frozen in liquid nitrogen, and 20 mg was placed in bead beater tubes (2 mL, CKMix, Bertin Technologies, France), and 10 μL of internal standard mixture (IS mix, 1 μM each) and 1 mL of a mixture containing methanol, water, and formic acid (70/25/5, v/v/v) was added. Homogenization and extraction were performed using a bead beater (Precellys Evolution homogenizer, Bertin Technologies, France) at 6000 rpm for 4 × 30 s with 20 s pauses between intervals. Subsequently, the samples were incubated for 12 h at −20 °C. Then, samples were centrifuged for 5 min at 13000 rpm (MiniSpin, Eppendorf), and the supernatant was transferred to a new microcentrifuge tube and freed of solvent under a stream of nitrogen. Samples were redissolved in water (20 μL, pH 2.5), acidified with hydrochloric acid (1 mM), and extracted twice with ethyl acetate (120 μL each). The combined organic layers were dried under nitrogen and subjected to derivatization with previously optimized parameters by adding an ethanolic EDC solution containing 6% pyridine (20 μL, 120 mM) and a GT solution (20 μL, 200 mM, in EtOH/H_2_O; 90/10; v/v) and shaking on a thermoshaker (25 °C, 600 rpm, 180 min). The derivatized samples were evaporated to dryness under nitrogen, resuspended in ACN/H_2_O (50/50, v/v, 50 μL), transferred to autosampler vials with inserts, and stored at −20 °C until the UHPLC-MS/MS analysis.

### Ultra-High Performance Liquid Chromatography-Triple Quadrupole Mass Spectrometry (UHPLC-MS/MS)

Mass spectrometry was conducted using a Shimadzu Nexera X2 UHPLC system (Shimadzu, Duisburg, Germany). The system comprised two pumps (LC-30AD), a degasser (DGU-20A5R), an autosampler (SIL-30AC), a column oven (CTO-30A) and a controller (CBM-20A). This UHPLC system was coupled to a QTRAP 6500 mass spectrometer (AB Sciex, Darmstadt, Germany) operated in ESI^+^ mode. The ion source parameters were set as follows: ion spray voltage at 4500 V, curtain gas at 35 psi, nebulizer gas at 55 psi, heater gas at 65 psi, collision-activated dissociation was set to “high”, and source temperature at 600 °C. Data acquisition was performed using Analyst 1.6.3 (AB Sciex, Darmstadt, Germany). For all reference compounds, individual MS/MS parameters were first tuned and optimized on the MS system, with two mass transitions recorded for each compound after derivatization (see Table S2, Supporting Information). Aliquots of samples (5 μL) were subjected to UHPLC-MS/MS analysis using an Acquity BEH C18 column (100 × 2.1 mm, 1.7 μm i.d.; Waters, Manchester, UK). Chromatographic separation was performed with a flow rate of 0.5 mL/min at 60 °C. Starting with 100% aqueous formic acid (0.1%) as eluent A and acetonitrile (0.1% formic acid) as eluent B, the content of B was kept isocratic for 0.5 min, then raised to 10% B within 0.5 min, increased to 30% B within 4 min, kept isocratic at 30% B for 2 min, increased to 80% B in 3 min and to 100% B in 0.1 min, and finally maintained at 100% B for 0.9 min. Column re-equilibration was achieved by maintaining the starting conditions (100% A) for 1 min after every run.

### Quantitative Analysis of Phytohormones by Means of UHPLC–MS/MS

Quantification was performed using a scheduled UHPLC-MS/MS method, where the specific mass transitions of all compounds were recorded within a 30 s window of each analyte’s retention time. The SIDA methodology was employed to determine the concentrations of phytohormones. A stock solution containing all analytes was spiked with deuterated standards at 19 different concentration ratios. By plotting the peak area ratios (analyte/internal standard) against the concentration ratios (analyte/internal standard), individual calibration curves for all compounds were established using linear regression (Table S3, Supporting Information).

### Validation Experiments

#### Limit of Detection (LOD), Limit of Quantification (LOQ), Recovery Rates, and Intraday/Interday Precision

LOD and LOQ were determined for each compound in an analyte-free chlorophyll matrix. Therefore, stock solutions containing all analytes were diluted successively and spiked with the chlorophyll matrix (0.1%, w/v). LOQ and LOD were calculated according to the literature.^45^ Recovery rates of all quantified compounds were determined in the analyte-free matrix and in tomato leaf matrix at different concentration levels (0.2, 2, 20, 200, and 2000 nM). The intraday precision was determined by comparing 3 individual UHPLC-MS/MS measurements within 1 day, and the interday precision was determined over 3 consecutive days (Table S4, Supporting Information).

*Matrix effects* (*ME*) were characterized as the ratio of the peak area of phytohormones spiked into analyte free chlorophyll matrix (0.1%, w/v) to the peak area of phytohormones in a standard solution. The ME values were calculated using the formula [(*A* – *B*)/*C* × 100%], where *A* denotes the peak area of phytohormones in the chlorophyll matrix spiked with reference standards, *B* represents the peak area of phytohormones in the nonspiked blank matrix sample, and C signifies the peak area of phytohormones in the reference standard solution (Table S4, Supporting Information).

### Nuclear Magnetic Resonance Spectroscopy (NMR)

Samples were analyzed on a Bruker AV III 400 MHz system (Bruker, Rheinstetten, Germany), which was equipped with a Z-gradient 5 mm multinuclear observe probe (BBFOplus) at a temperature of 298 K. NMR tubes used were 5 mm in diameter and 7 in. in length (Z107374 USC tubes, Bruker, Faellanden, Switzerland). Data processing was performed using Bruker TopSpin software (version 3.6). Manual adjustments were made for zero- and first-order phase correction, whereas baseline correction was performed automatically using the command ″absn.″ For qHNMR, the ERETIC 2 procedure was employed, as detailed in the literature.^42^ L-tyrosine (5.21 mmol/L) was used as the external standard for spectrometer calibration, specifically integrating the resonance signal at 7.1 ppm (m, 2H). Stock solutions of reference compounds were prepared, and their aliquots (600 μL each) were analyzed by qHNMR. The concentrations of all compounds were calculated using the ERETIC 2 software tool in TopSpin (Bruker, Rheinstetten, Germany) and subsequently used to prepare the calibration curves for UHPLC-MS/MS quantitation.

## Results and Discussion

Quantitative analysis of phytohormones using LC-MS is often limited to either highly abundant phytohormones that show good MS ionization responses or to samples available in large quantities, which may in turn require extensive sample cleanup and prefractionation. However, with the application of multifunctional chemical derivatization, it is possible to perform quantitative profiling of multiple phytohormone classes from low sample quantities. The current manuscript describes how effectively combining derivatization reagents led to the coverage of 27 phytohormones (Figure 1) from multiple compound classes in one UHPLC-MS/MS run within 10 min.

**Figure 1:**
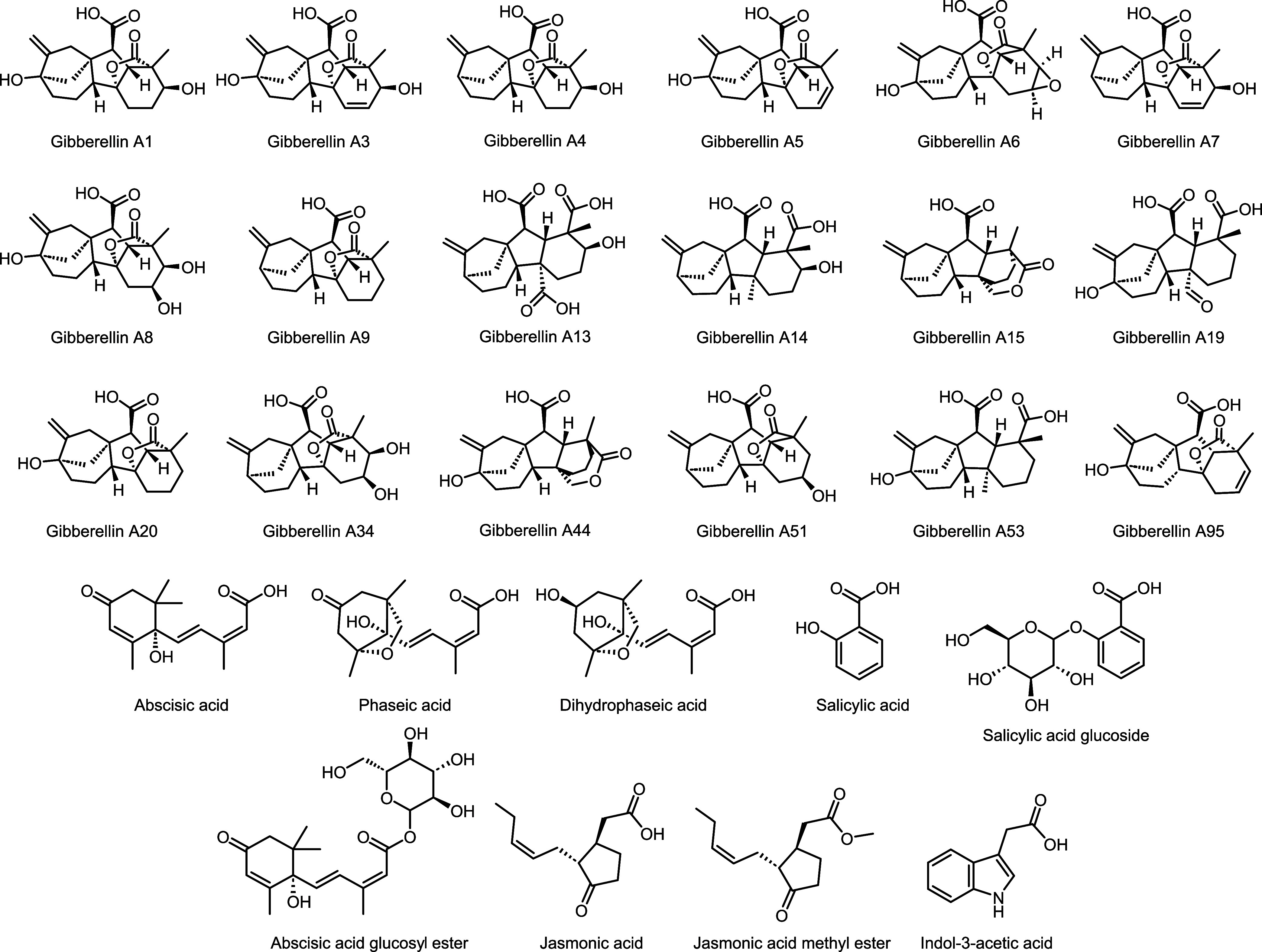
Chemical structures of phytohormones quantified using ultrahigh-performance liquid chromatography-tandem mass spectrometry in the MRM mode (UHPLC-MS/MS_MRM_).

### Multifunctional Chemical Derivatization Approach

To cover a broad range of phytohormones in one approach, different derivatization reagents were tested, focusing on their reactivity with carboxylic acids, commonly present in plant hormones. In particular, previous reports suggest that EDC yields good performance and sensitivity in the detection of GAs.^35,36,46^ GAs react with the carbodiimides, which are also used in the hydrazine derivatization reactions as an activator of carboxylic acids. However, GAs form stable derivatives with EDC via O→N migration instead of reacting with the hydrazine (Figure 2).

**Figure 2:**
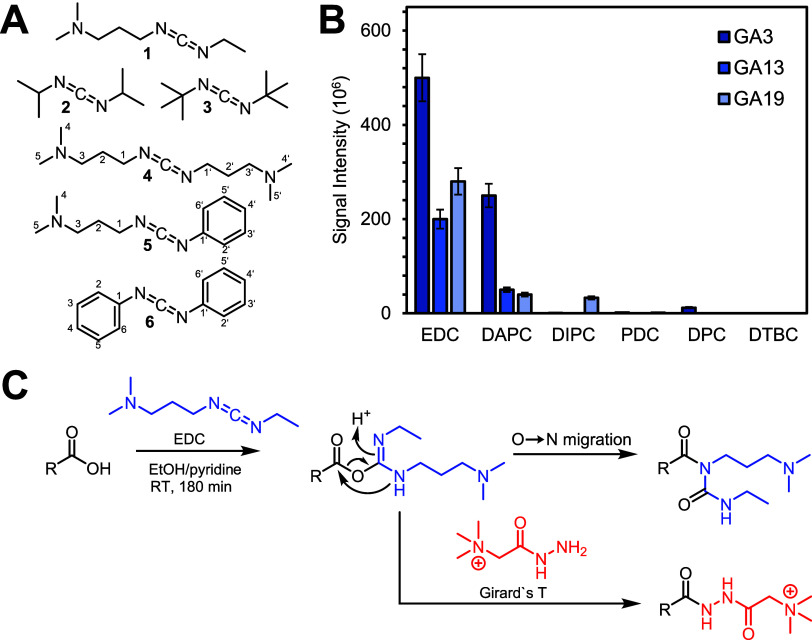
Carbodiimide reagents (1–6) evaluated regarding their effectiveness in the derivatization of phytohormones (A). Comparison of signal intensities (mean of triplicates ± SD) of gibberellin (GA) 3, GA13, and GA19 after derivatization with tested carbodiimides, leading to the selection of N-(3-(dimethylamino)­propyl)-N′-ethylcarbodiimide hydrochloride (EDC) as the most suitable reagent (B). Proposed multifunctional chemical derivatization strategy to facilitate highly sensitive detection of gibberellins (blue) and non-GA phytohormones (red) (C).

In the present study, this strategy was adopted, and different carbodiimides were screened to achieve optimal derivatization efficiencies. Moreover, the commercially available carbodiimides EDC (**1**), DIPC (**2**), and DTBC (**3**) and structurally similar analytes DAPC (**4**), PDC (**5**), and DPC (**6**) were synthesized via the respective urea compounds and tested for derivatization with a model mixture of GAs. The mixture of GA3, GA13, and GA19 was chosen as the representative for the mono-, di-, and tricarboxylate motifs encompassing the chemical diversity present within the GA compound class. Among the tested carbodiimides, EDC outperformed the others and was subsequently used for further optimization (Figure 2). Apart from GAs, the most promising candidate for hormone derivatization was GT, owing to its compatibility with EDC, and the introduction of a positively charged trimethylammonium moiety into the compounds of interest further boosted the MS response. To effectively derivatize GAs with EDC and non-GA hormones with GT, combinatorial derivatization was tested with the goal of combining both derivatization reactions in a one-pot approach (Figure 2). Mixtures of GAs and carbonyl-containing hormones were mixed with the reagents EDC and GT, and samples were analyzed using UHPLC-MS/MS to confirm the derivatization products and compare their absolute peak areas with individually derivatized samples. The combination of EDC and GT in the derivatization strategy proved successful, as using a mixture of the two reagents did not influence both GAs (as EDC derivatives) and non-GA hormones (as GT derivatives). Because EDC is commonly used in GT derivatization as an activator of carboxyl groups, the combination of both reagents worked well together, and combining EDC and GT made it possible to further increase the MS response of non-GA analytes. The finalized multifunctional chemical derivatization strategy involved the reaction of plant hormones with EDC and GT in ethanol with 6% pyridine as a catalyst. On the basis of previous reports, ethanol was chosen, and the GA derivatization with carbodiimides was intensively studied and optimized.^36^ Especially di- and tricarboxylated GAs showed lower derivatization response owing to intermediate anhydride formation, which could be partially resolved by alcohol (ethanol) addition, yielding the respective stable (ethyl) esters in the derivatization process (Figure S1).^46^

### Optimization of Derivatization Reaction Conditions

To achieve optimal derivatization of all analytes, the reaction conditions such as reaction temperature and time were optimized. The concentration of reagents was previously optimized and adapted for GT from the 3-nitrophenyl hydrazine (3-NPH) protocol published earlier.^43^ First, the derivatization temperature was optimized. Therefore, the reactions were performed at 25, 40, and 60 °C. Treatment at 10 °C was also tested to explore the potential of minimizing side reactions at below-ambient temperature. The signal areas of the derivatives decreased for GA3 and GA19 with increasing temperature. For GA13, an optimum was observed at 25 °C. Therefore, a derivatization temperature of 25 °C was established (Figure 3).

**Figure 3:**
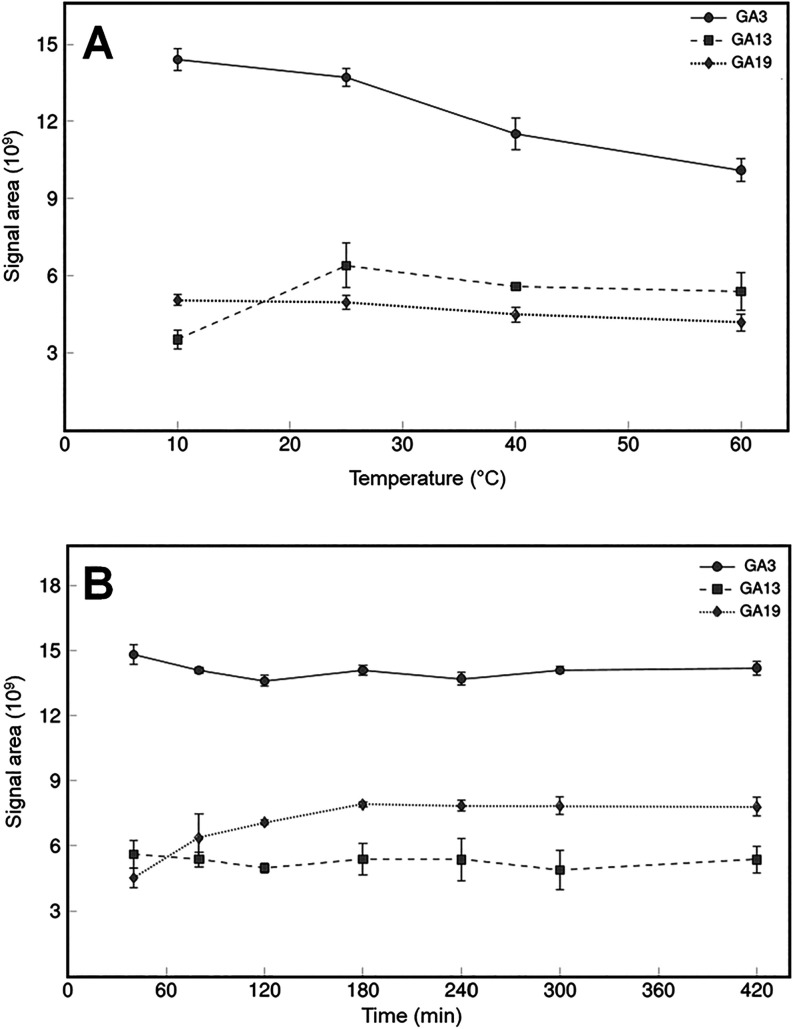
Optimization of reaction temperature (A) and reaction time (B) for N-(3-(dimethylamino)­propyl)-N′-ethylcarbodiimide hydrochloride (EDC)-based gibberellin (GA) derivatization with a GA mixture containing GA3, GA14, and GA19.

Next, the required derivatization time at the optimized derivatization temperature was investigated. The signal areas of the analytes GA3 and GA13 remained relatively constant over the analyzed periods ranging from 40 to 420 min. For GA19, an increase in the signal areas was observed up to 180 min. After this point, the height of the signal areas remained constant for all tested analytes. Therefore, the optimum derivatization time at 25 °C was determined to be 180 min (Figure 3). The rate-limiting step was the derivatization of the tricarboxylic acid-containing GA13, which showed no increase in peak areas after 180 min at 25 °C. The simultaneous derivatization of non-GA hormones was also completed within 180 min. If the analysis of tricarboxylic acid-containing GAs is of no interest, then the procedure can be shortened to 40 min. The final derivatization conditions were 120 mM EDC-HCl and 200 mM GT in EtOH with 6% pyridine at 25 °C for 180 min.

### Method Development and Validation Experiments

To facilitate the highly sensitive mass spectrometric detection of multiple phytohormone classes across a broad concentration range, samples were extracted using a mixture of methanol, water, and formic acid in the ratio of 75/20/5 (v/v), on the basis of previous reports.^35^ To achieve optimal extraction, a bead beater homogenizer was used instead of manually grinding the samples. The samples were equilibrated overnight at −20 °C and then centrifuged, and the supernatant was dried under nitrogen. Before derivatization, liquid–liquid extraction was performed using ethyl acetate and acidified water to further enrich the phytohormones. Then, the combined organic layers were derivatized with the previously optimized conditions before being subjected to UHPLC-MS/MS. To optimize the MS/MS detection, standard solutions of the analytes were derivatized and injected into the MS ion source via a syringe pump. Software-assisted adjustments to the ion source and ion path parameters were conducted in positive electrospray ionization mode to maximize ion abundance. Chromatographic separation of all 27 analytes was accomplished with the help of a BEH C18 column (Figure 4A).

**Figure 4:**
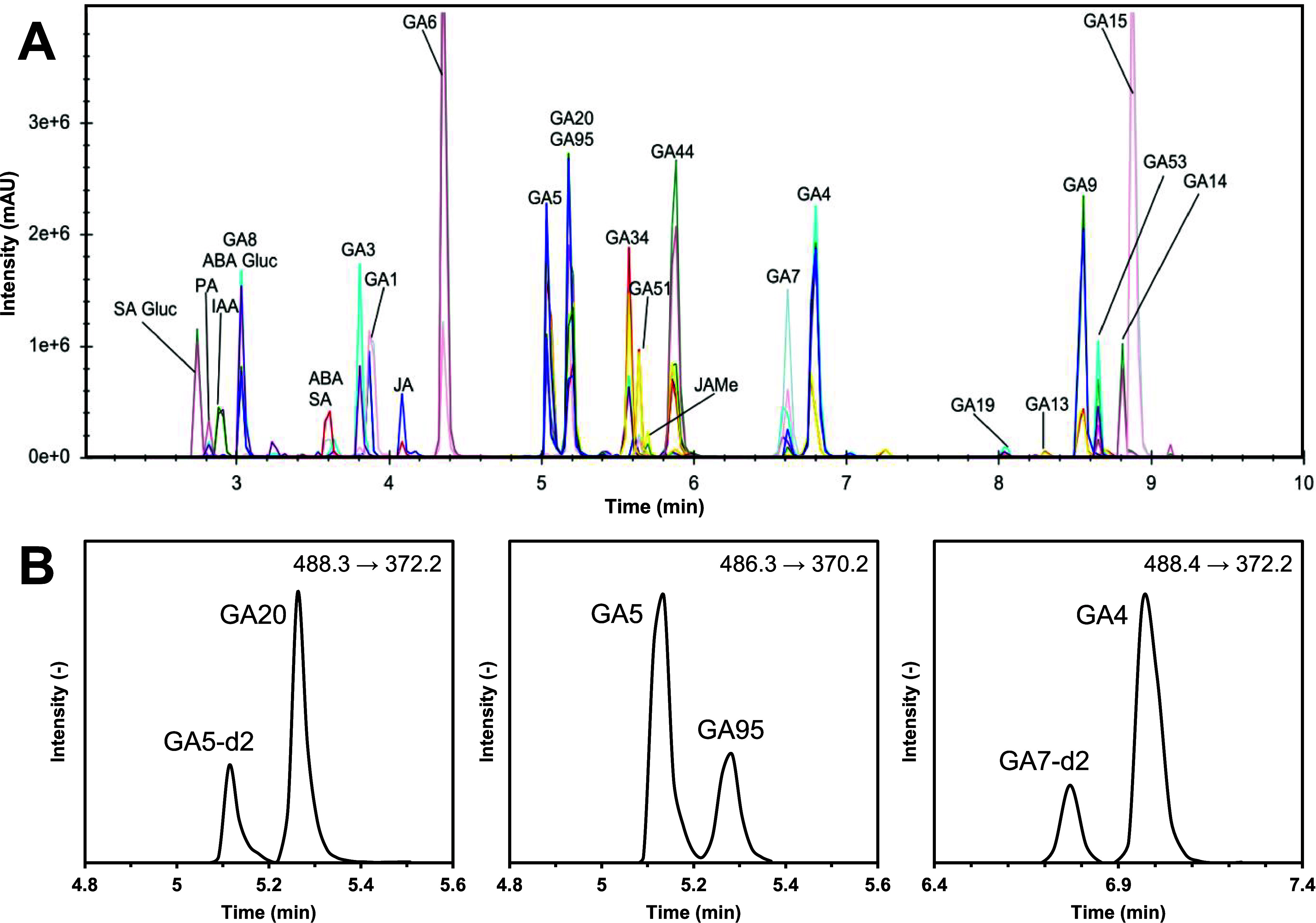
Ultrahigh-performance liquid chromatography-tandem mass spectrometry (UHPLC-MS/MS; ESI+) chromatograms in the scheduled MRM mode of derivatized phytohormones and deuterated internal standards used for quantitation using the stable isotope dilution assay (A). Chromatographic separation of isobaric analytes GA5-d2 and GA20 (B), GA5 and GA95 (C), GA 7-d2 and GA4 (D).

The BEH C18 column was specifically chosen to receive one peak per compound despite the formation of isomers during derivatization, as shown in Figure S1. Furthermore, with the chosen column, it was possible to chromatographically separate three pairs of isobaric analytes and internal standards that additionally showed identical Q1/Q3 mass transitions without the need for buffered solvents or pH adjustment. The near-baseline separation of the critical analytes GA20 and GA5-d2 (488.3 → 372.2 *m*/*z*), GA5 and GA95 (486.3 → 370.2 *m*/*z*), and GA4 and GA7-d2 (488.4 → 372.2 *m*/*z*) was achieved in less than 10 min (Figure 4B).

To verify the reliability of the method, a series of validation experiments were conducted in an analyte-free chlorophyll solution. The chlorophyll content of the matrix solution was adjusted to 0.1%, which approximately corresponds to the chlorophyll content in many edible plants.^47,48^ To demonstrate the general applicability of the extraction, derivatization, and LC-MS methods for plant samples, recovery experiments were conducted in triplicate in the chlorophyll matrix and, additionally, in tomato leaves to verify the accuracy and applicability in a real plant matrix. Therefore, four concentration levels ranging from 0.2 to 200 nM for GAs and from 20 to 2000 nM for non-GA hormones, owing to their higher abundance, were chosen. Most recovery rates were found to be in the range of 80–120% with a few exceptions (Figure S1). Di- and tricarboxylic acid-containing GAs showed lower recovery rates than monocarboxylate GAs, with recovery rates ranging from 61 to 92%. Moreover, GA13 and GA53 could not be detected at the lowest spike-in level of 0.2 nM, which is most likely due to the lower reaction yield resulting from intermediate anhydride formation and subsequent ethanolysis to corresponding ethyl esters, as discussed earlier (Figure S1). These issues have also been discussed in the literature, where di- and tricarboxylic acid-containing GAs were often not included in the analysis at all. Despite their lower recovery rates, the method achieves LODs in the nM range, which are essential to detect the physiological flux of gibberellins. Additionally, the consistent recovery rates across spiking experiments indicate that the method can reliably capture trends and support valid comparisons between genotypes or treatments, thereby enabling biological interpretations of metabolic changes, even for di- and tricarboxylic acid-containing GAs. With the present method, the detection of di- and tricarboxylic acid-containing GAs is possible, albeit with slightly higher limits of detection compared to monocarboxylic acids (Table S4). The recovery rates of most non-GA hormones were in the expected range, except for ABA-Gluc (76%), JA-Me (71%), and SA-Glc (134%). As no identical isotopically labeled internal standards are available for these hormone storage forms, the respective deuterated aglycons (ABA-*d*_6_ and SA-*d*_4_) or the labeled precursor (JA-*d*_5_) were used as internal standards. Potential differences in extraction and MS response could explain these deviations. However, the recovery rates did not differ drastically from the 80–120% range and were also mostly consistent over the spiking range of 0.2–200 nM for GAs and 20–2000 nM for non-GAs, which ensures reproducible analysis.

Furthermore, LODs and LOQs were established for all compounds on the basis of the signal-to-noise (S/N) ratios of the analytes, at S/N 3 for LOD and S/N 10 for LOQ. For this, calibration solutions were serially diluted in the chlorophyll matrix, extracted, derivatized, and analyzed by UHPLC-MS/MS. The obtained LODs were in the range of 0.01 nM (GA8 and GA53) to 8.96 nM (JA), while LOQs ranged from 0.04 nM (GA8 and GA53) to 29.9 nM (JA). All quantification limits (Table S3) were well within physiologically relevant concentrations; therefore, the derivatization method was deemed sufficient for phytohormone analysis. Additionally, intraday and interday variations were determined by measuring three calibration samples with added chlorophyll matrix within 1 day and three samples on three different days. By calculating the relative standard deviation (RSD) at a spiked concentration of 200 nM, the intraday precision ranged between 1.36 and 14.1%. Interday precision, measured on three consecutive days, ranged between 3.41 and 31.1%. As expected, higher values were observed for interday precision after storing the samples in the autosampler at 5 °C, as is usual for derivatized samples, suggesting that the measurements should be conducted immediately after derivatization. To prevent degradation, samples were frozen at −80 °C after derivatization, and measurements within 1 week did not show any sign of degradation. Because of the short run time of 10 min, one fully stocked autosampler (115 slots) can be assessed completely before sample degradation would influence the results. Matrix effects (ME) ranged from 70.5% to 115.9%, showing that the plant matrix only minimally affects the derivatization and ionization efficiency of the phytohormones during UHPLC-MS/MS analyses (Table S4, Supporting Information). The validation experiments conducted in this study were largely consistent with previously reported results.^35–37^ Some methods that reach even lower LODs often have other disadvantages, such as significantly longer run times, or limited analyte coverage, especially regarding GAs, or limited isotope-labeled internal standards.^31,35,49^ For example, Cai et al. monitored 14 GAs with recovery rates from 81–102% with LOQs of 0.5 nM in an 18 min LC-MS run time from 50 mg samples. Li et al. quantified 13 GAs with 64–107% recovery rates and a LOQ of 0.01 nM from <1 mg samples in 22 min LC-MS run time. In a one-pot approach, Cai et al. determined 31 hormones, with recovery rates of 80.7–120.4% and LOQs of 0.007–1.33 nM, in 18 min. Therefore, the present method offers a comparatively low LC-MS run time (10 min), high analyte coverage (18 GAs, 27 hormones total) from small sample amounts (20 mg), with comparable LOQs and recovery rates. Consequently, application of the present method to large-scale breeding studies benefits greatly from the short LC-MS run time while maintaining high analyte coverage and competitive LOQs. The derivatization procedure was then applied to various plant tissues.

### Quantitation of Phytohormones in Different Plant Samples via UHPLC-MS/MS

Compound quantification was realized using the newly developed SIDA methodology. Isotopically labeled internal standards were added to all samples before extraction and derivatization. Because not all analytes were available as labeled versions, structurally similar analogs were quantified with the most similar internal standards. For example, GA3 and GA14 were quantified using GA1-d_2_ and GA53-d_2_ as internal standards, respectively (*c*.*f*. Table S6 for details). For calibration, mixtures of reference compounds and internal standards were measured using UHPLC-MS/MS. By plotting the peak area ratios of the specific mass transitions against concentration ratios, the calibration curves were established, which exhibited a linear response with correlation coefficients of >0.99. Samples of important crop plants such as tomato and maize were chosen to test the applicability of the method to real plant material. Additionally, cress samples were chosen to elucidate the distribution of phytohormones in different plant tissues such as roots, seeds, stems, and leaves. Quantitative results revealed distinct differences between plant type and plant tissue (Figure 5).

**Figure 5:**
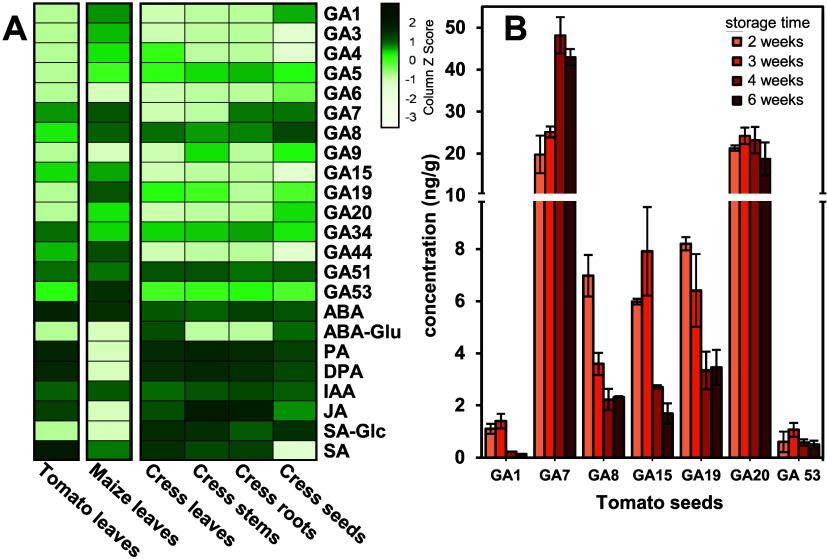
Heatmap displaying the quantitation results of phytohormones detected in samples of tomato, maize, and cress to assess the suitability of the developed method for different plant tissues (A). Comparison of major changes in gibberelline concentrations (mean of triplicates ± SD) during tomato seed storage for 2 to 6 weeks (B).

In the tomato leaf samples, the analytes GA7 and GA8 showed the highest concentrations. ABA and phaseic acid were detected at concentrations above 200 ng/g, whereas salicylic acid was present in concentrations of over 1000 ng/g. In comparison, several GAs were detected in maize leaves. The bioactive GAs GA1 and GA4 were present in concentrations of 5.23 and 1.00 ng/g, respectively. Notably, GA53 showed the highest concentration (96.5 ng/g). In cress, the GAs GA5, GA8, GA19, GA34, GA51, and GA53 could be detected in all parts of the plant, including cress seeds. The highest measured concentration of GA8 (110 ng/g) was found in cress stems. In the cress samples, GA1 could be quantified only in the seeds, with a concentration of 10.4 ng/g. GA51 was found in all three leaf matrices, with similar concentrations (8.0 ng/g) in tomato leaves and maize leaves, whereas an eight times higher concentration (32.2 ng/g) was detected in cress leaves. The concentrations of GAs in all samples ranged from approximately 0.1 to 30.0 ng/g, which is consistent with previously published data.^50,49^ Notably high concentrations were observed in maize leaf samples, specifically for GAs such as GA53 (96.5 ng/g), GA44 (25.3 ng/g), and GA1 (5.23 ng/g). The catabolites GA8, GA34, and GA51 were found in all matrices at relatively high concentrations. GA3 and GA14 are rarely found in plant metabolism and are primarily produced in fungi, and therefore, GA14 could not be detected in any samples.^7^ However, GA3 is produced in maize and plays a crucial role in seed germination.^51^ The concentration of GA3 observed in maize leaves falls within the range reported in the literature for maize seedlings.^51^ In terms of non-GA hormones, SA, ABA, IAA, JA, and SA-Glc were detected across all cress samples. An accumulation of JA was observed in the stems and roots of cress, with concentrations of 860 and 525 ng/g, respectively. In cress stems and leaves, JA was present at lower concentrations below 50 ng/g. ABA and IAA were detected in similar concentration ranges across the cress samples, which align with previous reports.^52^ ABA concentrations ranged from 13.2 to 71.8 ng/g, and IAA concentrations ranged from 18.4 to 57.9 ng/g. Cress leaves and stems showed high concentrations of SA and its glycosylated derivative SA-Glc (246 and 356 ng/g, respectively). In the cress leaves, a SA-Glc content of 216 ng/g was determined. Tomato seeds harvested from ripe tomato fruits were kept in storage for 2 to 6 weeks, and their phytohormone content was analyzed to monitor postharvest changes of gibberellin content, which is essential for assessing dormancy status, germination behavior, and seed quality. Among the detected GAs, the bioactive GA GA1 was primarily detected in seeds harvested early, and lower concentrations were detected in seeds stored for 4 and 6 weeks (Figure 5B). This trend was also observed for the GAs GA8, GA15, and GA19. For GA7, there was a higher concentration at shorter storage followed by a lower concentration at longer storage. GA53 was detected in relatively constant amounts in tomato seeds (Figure 5B). The concentration ranges of these plant hormones are consistent with previously reported values.^49,53–55^

In summary, in this study, a new highly specific, accurate, and robust SIDA-UHPLC–MS/MS method was developed and validated to simultaneously quantify 27 phytohormones in different plant tissues. Using a combination of hydrazine and carbodiimide reagents enabled the multifunctional chemical derivatization using a one-pot approach. The quantitative mapping of phytohormones will facilitate a better understanding of their dynamic regulation in response to abiotic and biotic stress, which could guide breeding programs in the future. Ultimately, the integration of plant breeding, molecular biology, and phytometabolomics will result in benefits along the value chain from producers to consumers by optimizing the yield and nutritional and sensory qualities of crops, leading to the production of sustainable, high-quality plant-based food products.

## Supplementary Material



## Notes

Peak area comparison of derivatization with and without GT addition, mass transitions of all analytes, calibration curves, determined recovery rates, method validation results, detailed derivatization mechanisms, and isotopically labeled standards used for SIDA (PDF)
