# Supplementary material for: Development of an Ultrahigh-Performance Liquid Chromatography–Triple Quadrupole Mass Spectrometry Method for Multiclass Phytohormone Quantification Via Multifunctional Chemical Derivatization
Source: J Agric Food Chem. 2026 May 18;74(20):15910–20. doi: 10.1021/acs.jafc.6c01953 (PMC13220312; doi:10.1021/acs.jafc.6c01953)
Supplement: Supplementary file 1 [file jf6c01953_si_001.pdf]

Supporting Information

Development of an ultra-high-performance liquid chromatography–triple quadrupole mass spectrometry method for multi-class phytohormone quantification via multifunctional chemical derivatization

Michael Gigl[1], Michael Dankesreiter[2], Larissa Barl[3], Carlos Agius[4], Christian Schmid [5], Magdalena Holzer[5], Chris-Carolin Schön[3], Claus Schwechheimer[4], Corinna Dawid[2,5,6*]

[1]Junior Research Group Food Processing and Health, ZIEL Institute for Food and Health, Technical University of Munich, Lise-Meitner-Str. 34, 85354 Freising, Germany

[2]Professorship for Chemosensory Food Systems, TUM School of Life Sciences, Technical University of Munich, Lise-Meitner-Str. 34, 85354 Freising, Germany

[3]Chair of Plant Breeding, TUM School of Life Sciences, Technical University of Munich, Liesel-Beckmann-Str. 2, 85354 Freising, Germany

[4]Chair of Plant Systems Biology, TUM School of Life Sciences, Technical University of Munich, Emil-Ramann-Strasse 8, Freising 85354, Germany

[5]Chair of Food Chemistry and Molecular Sensory Science, TUM School of Life Sciences, Technical University of Munich, Lise-Meitner-Str. 34, 85354 Freising, Germany

[6]Leibniz Institute for Food Systems Biology at the Technical University of Munich, Lise-Meitner-Str. 34, 85354 Freising, Germany

[*] To whom correspondence should be addressed

PHONE: +49-8161/71-2901

E-MAIL: corinna.dawid@tum.de

Table S1. Comparison of peak areas of phytohormone standard mixtures (200 nM each) derivatized with and without Girard's T addition.

Analyte	without GT		Peak Area with GT	
	Peak area[a]	Std. dev.	Peak area	Std. dev.
GA1	8.40E+06	6.64E+05	7.52E+06	1.82E+04
GA3	6.17E+06	7.14E+05	6.45E+06	4.75E+05
GA4	1.17E+07	9.16E+04	1.08E+07	5.72E+05
GA5	1.32E+07	5.01E+05	1.18E+07	1.10E+06
GA6	2.46E+07	2.31E+06	2.19E+07	5.42E+05
GA7	9.84E+06	6.57E+05	8.87E+06	1.12E+06
GA8	5.80E+06	1.70E+05	5.43E+06	2.72E+05
GA9	2.53E+07	1.47E+06	2.32E+07	1.12E+06
GA13	5.31E+04	2.01E+03	5.23E+04	4.69E+03
GA14	2.24E+07	6.52E+05	2.34E+07	2.04E+06
GA15	3.02E+07	3.17E+06	3.27E+07	1.90E+06
GA19	2.69E+06	1.09E+05	2.74E+06	1.10E+05
GA20	9.87E+06	4.42E+05	9.69E+06	1.62E+06
GA34	8.05E+06	1.07E+06	7.11E+06	2.89E+05
GA44	2.18E+07	5.92E+05	2.24E+07	1.42E+06
GA51	8.73E+05	5.03E+04	9.15E+05	4.01E+04
GA53	9.01E+06	6.98E+05	9.40E+06	6.22E+05
GA95	9.69E+06	2.25E+05	9.37E+06	5.86E+05
ABA	-		1.25E+06	1.21E+05
ABA-Glc	-		1.56E+05	1.45E+03
IAA	-		7.63E+05	4.40E+04
JA	-		3.57E+05	1.42E+04
JA-Me	-		6.65E+06	2.58E+05
DPA	-		1.09E+05	1.12E+04
PA	-		1.11E+05	1.06E+04
SA-Glc	-		3.81E+05	3.25E+04
SA	-		8.35E+05	1.25E+05

[a]Average peak area of 3 replicates.

Table S2. Mass transitions of derivatized phytohormones and internal standards used for quantification via UHPLC-MS/MS_{MRM}.

Analyte[a,b]	Q1 (m/z)	Q3 (m/z)	CE (V)	DP (V)	CE (V)	CXP (V)
GA1-d4 quant.	508.4	392.1	10	76	47	42
GA1-d4 qual.	508.4	437.3	10	76	33	50
GA4-d2 quant.	490.4	374.2	10	76	45	40
GA4-d2 qual.	490.4	419.2	10	76	31	48
GA5-d2 quant.	488.4	372.2	10	66	43	40
GA5-d2 qual.	488.4	417.1	10	66	31	46
GA6-d2 quant.	504.4	388.1	10	46	47	42
GA6-d2 qual.	504.4	433.3	10	46	31	20
GA7-d2 quant.	488.4	417.3	10	61	29	48
GA7-d2 qual.	488.4	372.2	10	61	39	40
GA8-d2 quant.	522.4	406.2	10	46	47	44
GA8-d2 qual.	522.4	451.3	10	46	31	22
GA9-d2 quant.	474.4	358.2	10	31	45	40
GA9-d2 qual.	474.4	403.4	10	31	29	46
GA19-d2 quant.	548.4	301.1	10	51	33	36
GA19-d2 qual.	548.4	273.1	10	51	37	30
GA20-d2 quant.	490.4	374.2	10	66	45	42
GA20-d2 qual.	490.4	419.3	10	66	29	20
GA34-d2 quant.	506.4	390.2	10	41	47	42
GA34-d2 qual.	506.4	435.3	10	41	31	22
GA44-d2 quant.	504.4	433.3	10	41	31	22
GA44-d2 qual.	504.4	388.2	10	41	47	42
GA51-d2 quant.	490.4	374.2	10	71	43	40
GA51-d2 qual.	490.4	419.2	10	71	29	48
GA53-d2 quant.	534.4	259.2	10	71	37	30
GA53-d2 qual.	534.4	287.2	10	71	29	34
GA1 quant.	504.4	388.2	10	81	45	42
GA1 qual.	504.4	433.3	10	81	31	22
GA3 quant.	502.4	431.3	10	71	31	22
GA3 qual.	502.4	386.1	10	71	41	42
GA4 quant.	488.4	372.2	10	81	45	40
GA4 qual.	488.4	417.2	10	81	31	48
GA5 quant.	486.4	370.2	10	66	45	40
GA5 qual.	486.4	415.2	10	66	29	48
GA6 quant.	502.4	386.2	10	61	47	42
GA6 qual.	502.4	431.2	10	61	31	48
GA7 quant.	486.4	415.2	10	46	29	20
GA7 qual.	486.4	370.2	10	46	41	42

GA8 quant.	520.4	404.2	10	41	47	44
GA8 qual.	520.4	449.2	10	41	33	22
GA9 quant.	472.4	356.1	10	66	45	40
GA9 qual.	472.4	401.2	10	66	29	46
GA13 quant.	516.4	445.3	10	26	33	50
GA13 qual.	516.4	400.3	10	26	49	42
GA14 quant.	532.4	174.2	10	51	31	20
GA14 qual.	532.4	129.1	10	51	39	14
GA15 quant.	486.3	415.3	10	16	33	46
GA15 qual.	486.3	370.2	10	16	47	42
GA19 quant.	546.3	299.1	10	21	35	34
GA19 qual.	546.3	271.1	10	21	35	34
GA20 quant.	488.3	372.2	10	71	45	42
GA20 qual.	488.3	417.2	10	71	31	46
GA34 quant.	504.3	388.2	10	56	45	42
GA34 qual.	504.3	433.1	10	56	33	48
GA44 quant.	502.4	431.1	10	81	31	20
GA44 qual.	502.4	386.2	10	81	51	42
GA51 quant.	488.3	372.2	10	36	45	38
GA51 qual.	488.3	417.1	10	36	29	22
GA53 quant.	532.3	257.1	10	56	41	28
GA53 qual.	532.3	285.2	10	56	31	34
GA95 quant.	486.3	115.3	10	51	20	16
GA95 qual.	486.3	370.3	10	51	41	48
ABA-d6 quant.	384.3	100.1	10	56	33	12
ABA-d6 qual.	384.3	132.0	10	56	35	14
IAA-d5 quant.	291.2	100.1	10	46	25	12
IAA-d5 qual.	291.2	132.1	10	46	41	16
JA-d5 quant.	329.3	100.1	10	21	33	4
JA-d5 qual.	329.3	117.9	10	21	33	36
SA-d4 quant.	280.2	235.2	10	41	27	26
SA-d4 qual.	280.2	85.9	10	41	27	10
ABA quant.	378.2	100.1	10	86	33	12
ABA qual.	378.2	132.1	10	86	35	16
PA quant.	394.2	100.1	10	51	35	14
PA qual.	394.2	132.1	10	51	39	34
DPA quant.	396.2	100.1	10	51	35	14
DPA qual.	396.2	132.1	10	51	39	34
ABA-Gluc quant.	540.3	378.1	10	101	39	22
ABA-Gluc qual.	540.3	60.1	10	101	115	18
IAA quant.	289.1	100.0	10	41	25	14

IAA qual.	289.1	130.0	10	41	41	14
JA quant.	324.2	100.0	10	56	31	12
JA qual.	324.2	117.9	10	56	33	14
JA-Me quant.	338.2	279.2	10	86	25	26
JA-Me qual.	338.2	205.2	10	86	31	22
SA quant.	276.1	231.1	10	36	21	30
SA qual.	276.1	86.1	10	36	23	10
SA-Gluc quant.	456.1	223.1	10	121	27	26
SA-Gluc qual.	456.1	121.0	10	121	71	14

[a]Quantifier mass transition used for quantification.
[b]Qualifier mass transition used for identification.

Table S3. Calibration curves of individual analytes obtained by plotting peak area ratios against concentration ratios using linear regression (y = m × x + t).

Analyte	m	t	R^2
GA1	0.004020	0.000127	99.97
GA3	0.001970	0.000667	99.97
GA4	0.003540	-0.000259	99.99
GA5	0.003700	0.010040	99.99
GA6	0.008720	0.002050	99.96
GA7	0.003190	0.000834	99.95
GA8	0.004130	0.005710	99.99
GA9	0.004640	0.001210	99.96
GA13	0.000125	0.001040	99.99
GA14	0.008530	0.000029	99.97
GA15	0.009430	0.001250	99.96
GA19	0.012280	0.003200	99.85
GA20	0.003140	0.000173	99.99
GA34	0.002200	0.006160	99.99
GA44	0.003920	0.001010	99.99
GA51	0.000263	0.008020	99.99
GA53	0.003710	0.001490	99.99
GA95	0.001880	-0.000110	99.99
ABA	0.005720	0.001990	99.91
ABA-Gluc	0.000394	0.000986	99.22
IAA	0.004370	0.020770	99.57
JA	0.002290	0.010560	99.93
JA-Me	0.052470	-0.003340	99.34
PA	0.000493	0.000996	99.53
DPA	0.000411	0.001045	99.94
SA-Gluc	0.006510	0.003740	99.12
SA	0.004830	0.073170	99.72

Figure S1. Recovery rates of gibberellins after multifunctional chemical derivatization and UHPLC-MS/MS quantification at the indicated spiking level in a 0.1% chlorophyll matrix (A) and spiked into a tomato leaf sample (B).

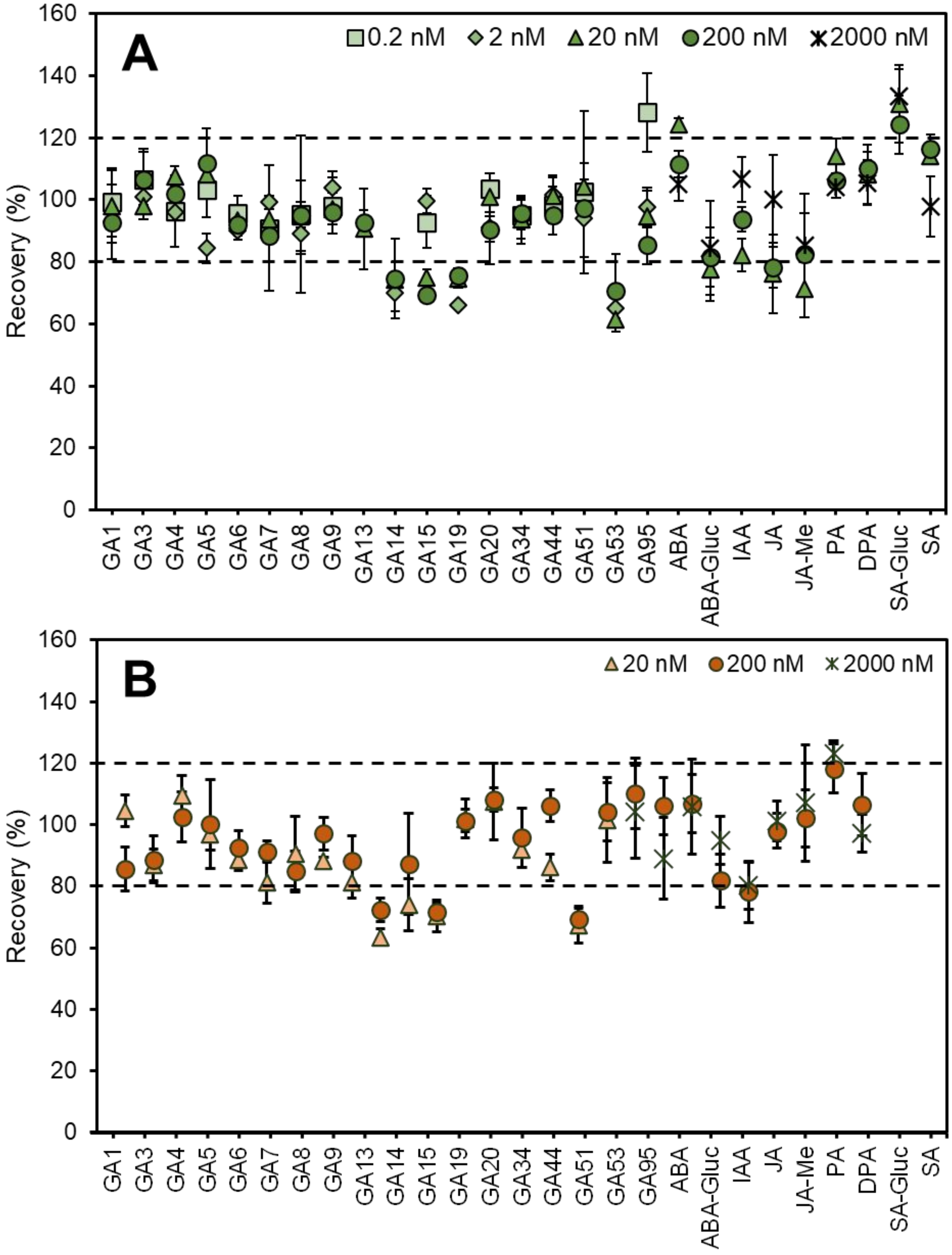

Table S4. Results of method validation experiments.

Analyte	LOD (S/N 3) [nM]	LOQ (S/N 10) [nM]	Intraday [%]	Interday [%]	Matrix Effect [%]
GA1	0.10	0.32	12.59	13.84	93.1
GA3	0.08	0.27	5.03	13.40	109.2
GA4	6.00	20.0	1.36	8.02	92.2
GA5	0.06	0.21	12.17	12.89	107.0
GA6	0.02	0.08	12.17	14.48	95.1
GA7	0.24	0.80	5.80	6.19	105.5
GA8	0.01	0.04	10.11	22.45	101.5
GA9	0.23	0.77	7.37	9.94	105.0
GA13	6.00	20.0	12.31	21.28	81.5
GA14	0.07	0.24	12.36	25.24	78.8
GA15	0.12	0.40	7.64	17.34	72.0
GA19	0.02	0.07	4.74	16.25	78.3
GA20	6.00	20.0	2.62	7.24	98.9
GA34	0.26	0.87	2.02	6.84	99.3
GA44	0.07	0.25	2.24	3.41	88.9
GA51	0.03	0.11	13.09	10.85	75.1
GA53	0.01	0.04	7.21	24.41	70.5
GA95	0.86	2.86	3.81	4.15	90.8
ABA	0.76	2.53	2.43	20.30	112.0
ABA-Glc	6.67	22.20	9.47	20.04	76.0
IAA	1.23	4.11	6.27	16.69	106.0
JA	8.96	29.90	12.01	17.96	111.7
JA-Me	7.32	24.40	14.11	31.12	81.5
DPA	1.64	5.46	6.71	13.35	99.8
PA	2.17	7.22	4.28	9.46	88.0
SA-Glc	3.70	12.30	2.43	14.30	115.9
SA	3.24	10.80	4.68	7.58	93.1

Figure S2. Mechanism of Gibberellins reacting with EDC, indicating the formation of two isomers (A), and the reaction pathway leading to the ethyl esters of dicarboxy-GAs via intermediate anhydride formation and subsequent esterification with ethanol according to Li et al (2016).

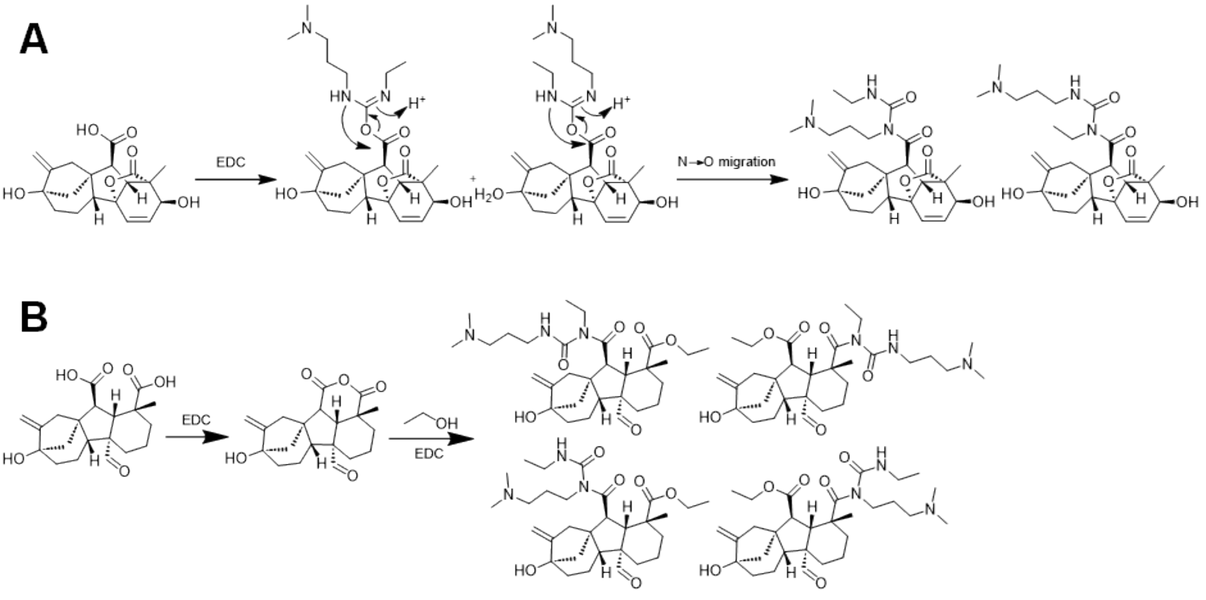

Table S5. Analytes and the isotopically labeled internal standards used for quantitation.

Analyte	Internal Standard
GA1	GA1-d4
GA3	GA1-d4
GA4	GA4-d2
GA5	GA5-d2
GA6	GA6-d2
GA7	GA7-d2
GA8	GA8-d2
GA9	GA9-d2
GA13	GA19-d2
GA14	GA53-d2
GA15	GA9-d2
GA19	GA19-d2
GA20	GA20-d2
GA34	GA34-d2
GA44	GA44-d2
GA51	GA51-d2
GA53	GA53-d2
GA95	GA20-d2
ABA	ABA-d6
ABA-Glc	ABA-d6
IAA	IAA-d2
JA	JA-d5
JA-Me	JA-d5
DPA	ABA-d6
PA	ABA-d6
SA-Glc	SA-d4
SA	SA-d4

References

Li, D.; Guo, Z.; Chen, Y. Direct Derivatization and Quantitation of Ultra-trace Gibberellins in Sub-milligram Fresh Plant Organs, Mol. Plant. 2016, 9, pp. 175–177.
